# Fabrication of (Ge_0.42_Sn_0.58_)S Thin Films via Co-Evaporation and Their Solar Cell Applications

**DOI:** 10.3390/ma17030692

**Published:** 2024-02-01

**Authors:** Daiki Motai, Hideaki Araki

**Affiliations:** National Institute of Technology (KOSEN), Nagaoka College, Niigata 940-8532, Japan

**Keywords:** SnS, (Ge, Sn)S, solid solution, co-evaporation method

## Abstract

In this study, as a novel approach to thin-film solar cells based on tin sulfide, an environmentally friendly material, we attempted to fabricate (Ge, Sn)S thin films for application in multi-junction solar cells. A (Ge_0.42_ Sn_0.58_)S thin film was prepared via co-evaporation. The (Ge_0.42_ Sn_0.58_)S thin film formed a (Ge, Sn)S solid solution, as confirmed by X-ray diffraction (XRD) and Raman spectroscopy analyses. The open circuit voltage (*V*_oc_), short circuit current density (*J*_sc_), fill factor (*FF*), and power conversion efficiency (*PCE*) of (Ge_0.42_ Sn_0.58_)S thin-film solar cells were 0.29 V, 6.92 mA/cm^2^, 0.34, and 0.67%, respectively; moreover, the device showed a band gap of 1.42–1.52 eV. We showed that solar cells can be realized even in a composition range with a relatively higher Ge concentration than the (Ge, Sn)S solar cells reported to date. This result enhances the feasibility of multi-junction SnS-system thin-film solar cells.

## 1. Introduction

Developing energy resources to replace fossil fuels is an important goal of current energy research. Photovoltaic (PV) power generation is a promising technology for sustainable energy production. As the demand for renewable energy increases, there is a need to increase the number of solar cell installations and to fabricate higher efficiency solar cells. To increase the number of solar cell installations, solar cells need to be light and flexible enough to be installed in previously impossible places, such as on curved surfaces or delicate buildings. This can be achieved by making the material thinner and forming it on a flexible substrate; therefore, compound thin-film semiconductors that can sufficiently absorb light are attracting research attention. Multi-junction compound solar cells consisting of materials with different band gaps have been widely studied and demonstrated to absorb sunlight efficiently. The light-absorbing layers of such multi-junction solar cells should exhibit adjustable bandgaps. Thin-film solar cells fabricated with one such material, Cu(In,Ga)Se_2_ (CIGS), exhibited a power conversion efficiency of over 23% [1]. However, CIGS contains several rare metals, such as the toxic elements Se, In, and Ga, and the resource constraints and environmental impact of its mass production are concerning. It is therefore vital to develop new alternative materials. Thus, this study focuses on a compound semiconductor—a tin sulfide (SnS)-based compound—which can be an alternative to CIGS regarding resources, cost, and environment.

SnS is a binary chalcogenide compound composed of tin and sulfur. It shows high potential as an environmentally friendly material because it is composed of low-toxicity elements abundant in the Earth’s crust. The structural polymorphism of SnS at room temperature is known to be orthorhombic SnS (Pnma), rock salt SnS (P213), and sphalerite SnS (F-43 m) [2,3,4,5]. This study focuses on the most studied form, orthorhombic SnS. Note that from here on, orthorhombic SnS is denoted as SnS.

SnS thin films can be prepared by various methods, including thermal evaporation [6,7], co-evaporation [8,9,10], sputtering [11,12,13,14], sulfurization [15,16], atomic layer deposition [17,18,19], and chemical bath deposition [20]. This indicates that SnS has a high degree of freedom in fabrication methods. In addition, SnS exhibits a band gap of 1.3 eV [8,9,15,21,22,23] and a high absorption coefficient [21,23]. This means that SnS is suitable for use in the light-absorbing layers of solar cells.

The development history of solar cells using SnS is described briefly below. In 1994, Noguchi et al., produced the first solar cell (ITO/n-CdS/p-SnS/Ag structure) using a SnS film and reported that it exhibited photovoltaic properties [21]. In 2006, Reddy fabricated a SnS thin-film solar cell with a SnO_2_/p-SnS/CdS/ITO structure and showed a *PCE* of 1.3% [24]. In 2013, Ikuno et al. fabricated a super straight type SnS thin-film solar cell with an ITO/Zn_1−*x*_Mg*_x_*O/p-SnS/Cu structure and reported a conversion efficiency of 2.1% [25]. A year later, Sinsermsuksakul et al., fabricated a substrate-type Mo/p-SnS/SnO_2_/Zn(O,S):N/ZnO/ITO structure cell that showed a conversion efficiency of 4.36% [18], significantly improving the performance of SnS thin-film solar cells. Despite the steady improvement in conversion efficiency, it has stagnated in the 4% range [26,27], and research on solar cell applications of SnS is at a low ebb. However, novel approaches have been recently investigated for developing SnS solar cell applications. Here, we present two state-of-the-art approaches for SnS solar cells.

The first is the preparation of SnS with n-type conduction and its application to homojunction solar cells. SnS usually exhibits p-type conduction because tin vacancies (V_sn_) in the sulfur-rich state and tin sulfur antisite (Sn_S_) in the tin-rich state behave as acceptor-type defects [13,28]. Yanagi et al. succeeded in preparing bulk SnS with n-type conduction by doping Cl [29], and Suzuki et al. reported that n-type SnS thin films can be prepared by supplying sulfur plasma while sputtering Cl-doped SnS [13]. Furthermore, Kawanishi et al. have achieved the fabrication of homojunction solar cells by employing p-type SnS thin films and n-type SnS single crystals [14].

The second is the preparation of SnS with a bandgap tunable for application to multi-junction solar cells. Regarding fabricating high-efficiency solar cells, multi-junction compound solar cells consisting of materials with different band gaps have been widely studied and were demonstrated to absorb sunlight efficiently. The light-absorbing layers of such multi-junction solar cells should exhibit adjustable bandgaps. Very recently, Kawamura et al. have succeeded in broadening the bandgap of SnS to ~2.25 eV by solid-dissolving alkaline earth metals in SnS [30].

Thus, SnS solar cells have again attracted attention in recent years due to novel approaches that take advantage of their potential capabilities.

In this study, as an approach to wide-bandgap SnS, we attempted to fabricate a (Ge, Sn)S thin-film solar cell by dissolving Ge in SnS. (Ge, Sn)S, a solid solution of SnS and germanium sulfide (GeS), shows a tunable band gap (between 1.3 and 1.6 eV) in its nanocrystals depending on the Ge concentration [31]. However, there have been very few reports on fabricating (Ge, Sn)S thin films; therefore, this topic requires further investigation. A previous study reported the fabrication of (Ge*_x_* Sn_1−*x*_)S (*x* = 0.18, 0.27) thin films via co-evaporation and their application in thin-film solar cells [32]. Our study reports the fabrication and solar cell applications of (Ge*_x_* Sn_1−*x*_)S thin films containing more Ge than previously reported. We then discuss the Ge solid solution effect by comparing it with the SnS we fabricated in the past [32].

## 2. Materials and Methods

Molybdenum disks (Mo, 99.95%, 3-mm thick, Furuuchi Chemical Corporation, Tokyo, Japan), tin shots (99.9999%, Furuuchi Chemical Corporation, Tokyo, Japan), germanium shots (99.999%, Furuuchi Chemical Corporation, Tokyo, Japan), sulfur chunks (S, 99.9999%, Furuuchi Chemical Corporation, Tokyo, Japan), cadmium iodide (CdI_2_, 99%, FUJIFILM Wako Pure Chemical Corporation, Osaka, Japan), thiourea (CS(NH_2_)_2_, 98%, Nacalai Tesque Inc., Kyoto, Japan), ammonia water (NH_3_, 28 wt%, FUJIFILM Wako Pure Chemical Corporation, Osaka, Japan), ZnO: Al (Al_2_O_3_ = 2 wt%, 3-mm thick, 99.99%, Furuuchi Chemical Corporation, Tokyo, Japan), and aluminum (Al, 99.99%, The Nilaco Corporation, Tokyo, Japan) were used as purchased.

First, approximately 0.8 μm Mo was deposited as the bottom electrode on an ultrasonically cleaned Eagle XG (Corning, Corning, NY, USA) substrate using DC sputtering (deposition time; 25 min, applied current; 1.0 A, process pressure; 0.4 Pa, Ar flow; 20 sccm). The deposition was performed under the same sputtering conditions under which the Mo thin film with a resistivity of 3.6 × 10^−5^ ohm/cm was obtained. Subsequently, SnS and (Ge*_x_*Sn_1−*x*_)S thin films were deposited by the co-evaporation of Ge, Sn, and S at pressures on the order of 10^−4^ Pa for 3 h using independent deposition sources. Sulfur is typically supplied as S_8_ molecules; however, to ensure a higher supply of reactive sulfur, S_8_ molecules were decomposed into S*_x_* by thermal cracking at 800 °C. Table 1 lists the manufacturing conditions in detail.

(Ge*_x_* Sn_1−*x*_)S thin films were fabricated under lower substrate temperature conditions (150 °C) than those used to synthesize SnS thin films to suppress the re-evaporation of GeS (which exhibits a high vapor pressure).

**Figure 1:**
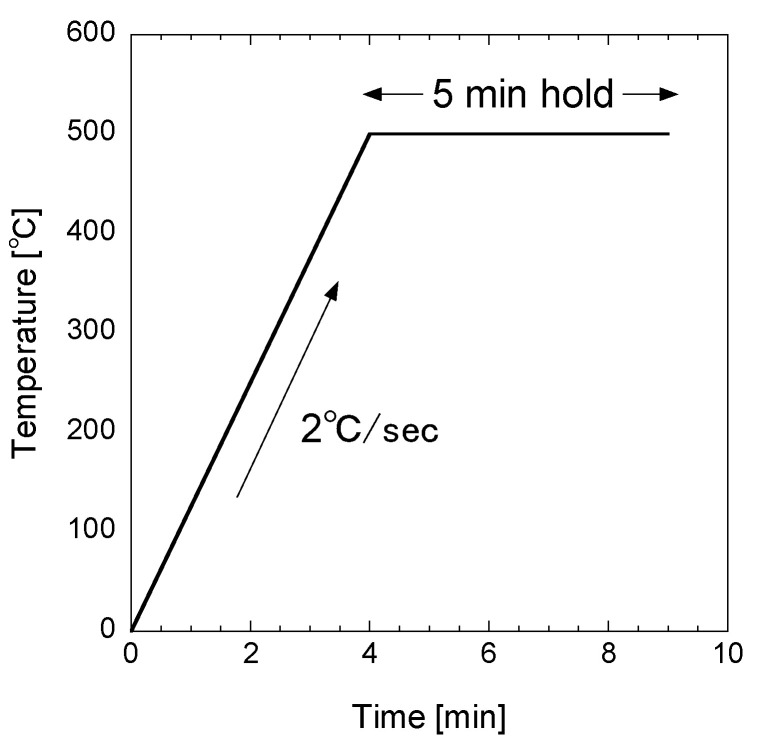
Temperature profile during the annealing of (Ge*_x_* Sn_1−_*_x_*)S thin films.

The as-prepared (Ge*_x_* Sn_1−*x*_)S thin films were annealed under a nitrogen atmosphere (the process pressure was atmospheric pressure, 1 × 10^5^ Pa) in an infrared heating furnace. The temperature profile during annealing is shown in Figure 1. The fabricated thin films were analyzed using X-ray fluorescence spectroscopy (XRF, ZSX Primus IV, Rigaku Corporation, Tokyo, Japan), X-ray diffraction (XRD, MiniFlex, Rigaku Corporation, Tokyo, Japan), and Raman spectroscopy (RMP-510, JASCO Corporation, Tokyo, Japan) at an excitation wavelength of 532 nm, and scanning electron microscopy (SEM, JSM-6060LV, JEOL Ltd., Tokyo, Japan).

Subsequently, an n-type CdS buffer layer approximately 90 nm thick was deposited on the (Ge*_x_* Sn_1−*x*_)S absorption layer by the CBD method at 70 °C for 20 min using a solution consisting of cadmium iodide (CdI_2_; 3.58 mmol L^−1^), ammonia water (NH_3_; 2.83 mol L^−1^), and thiourea ((NH_2_)_2_CS); 0.298 mol L^−1^). The fabricated (Ge*_x_* Sn_1−*x*_)S /CdS thin films were annealed in air at 200 °C for 20 min [33]. Next, Al-doped zinc oxide (ZnO:Al) was deposited as a window layer by radio frequency sputtering (deposition time; 110 min, RF power; 100 W, process pressure; 0.5 Pa, Ar flow; 20 sccm). The deposition was performed under the same sputtering conditions under which the ZnO:Al thin film was obtained with a thickness of approximately 450 nm, a resistivity of 1 × 10^−4^ ohm/cm, and a transmittance of >80% in the visible light wavelength range 400–1000 nm. Finally, Al was deposited as the top electrode by thermal evaporation.

The final thin-film solar cells with an Eagle XG/Mo/SnS or (Ge*_x_* Sn_1−*x*_)S/CdS/ZnO:Al/ Al structure were scribed into cells with an approximate size of 4.4 mm × 4.4 mm (aperture area ~0.16 cm^2^). The photovoltaic characteristics of the fabricated solar cells were evaluated using a solar simulator (SX-UI 500XQ, Ushio Inc., Tokyo, Japan) under AM 1.5 and 100 mW/cm^2^ irradiation. The external quantum efficiency (EQE) of the fabricated solar cells was evaluated using a solar cell evaluation system (SML-250 J, Bunkoukeiki Co., Ltd., Tokyo, Japan).

## 3. Results and Discussion

The elemental compositions of the fabricated SnS and (Ge*_x_* Sn_1−*x*_)S thin films were analyzed by XRF, as summarized in Table 2. The average composition in a φ 20-mm diameter plane near the center of a 25 mm × 25 mm sample was evaluated, and a summary of the compositional ratios is given. The composition of the thin films was estimated using analysis software (ZSX Primus IV, Rigaku Co., Tokyo, Japan) based on the fundamental parameter method. The error in the composition values of Sn, Ge, and S is less than 1% in repeated measurements of the same sample. The SnS film was thinner than the (Ge*_x_* Sn_1−*x*_)S film, possibly due to the re-evaporation of SnS during fabrication, which was carried out at a substrate temperature of 300 °C. The Ge/(Ge + Sn) composition ratio of the (Ge*_x_* Sn_1−*x*_)S thin film was estimated to be 0.42; the thin film has been subsequently denoted as (Ge_0.42_ Sn_0.58_)S. For the (Ge_0.42_ Sn_0.58_)S thin film, annealing decreased the film thickness and increased the (Ge + Sn)/S composition ratio, although its Ge/(Ge + Sn) composition ratio remained unchanged. This could be attributed to the re-evaporation of sulfur during annealing. The S-rich composition compared to stoichiometric composition was confirmed for both SnS and (Ge_0.42_ Sn_0.58_)S. According to a previously published first-principles study, S-rich SnS is more likely to form tin vacancy (V_sn_) defects at shallow energy levels than sulfur vacancy (V_s_) and sulfur antisite tin (Sn_S_) defects at deep energy levels [34]. Therefore, it is speculated that the fabricated SnS thin film had relatively fewer Vs, which can serve as recombination centers, and a thin film with excellent potential was fabricated.

**Figure 2:**
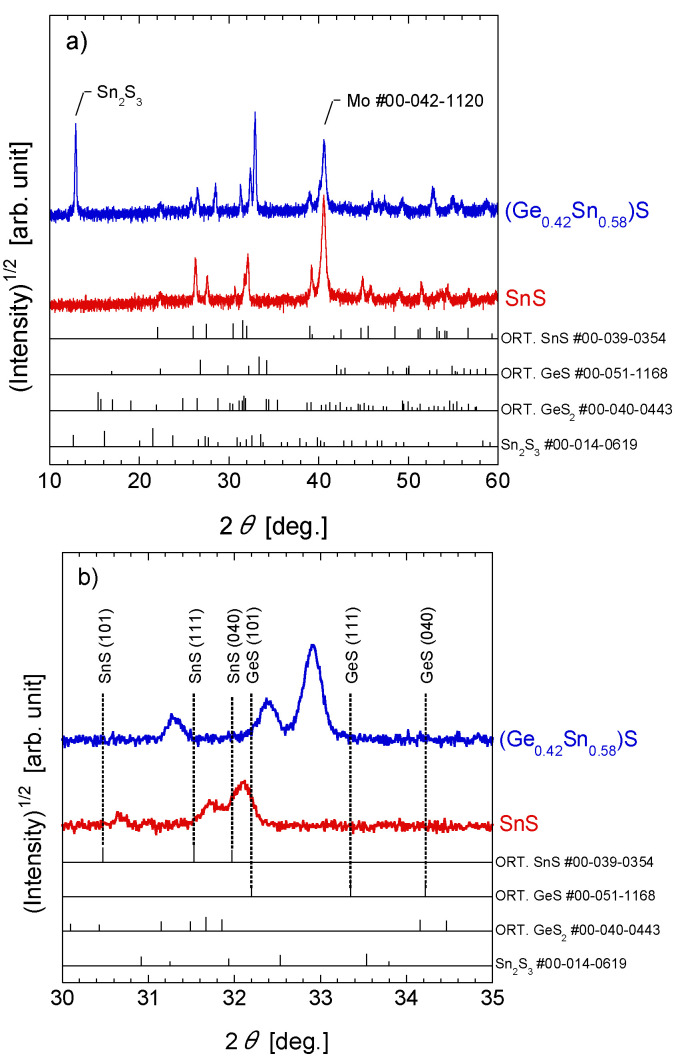

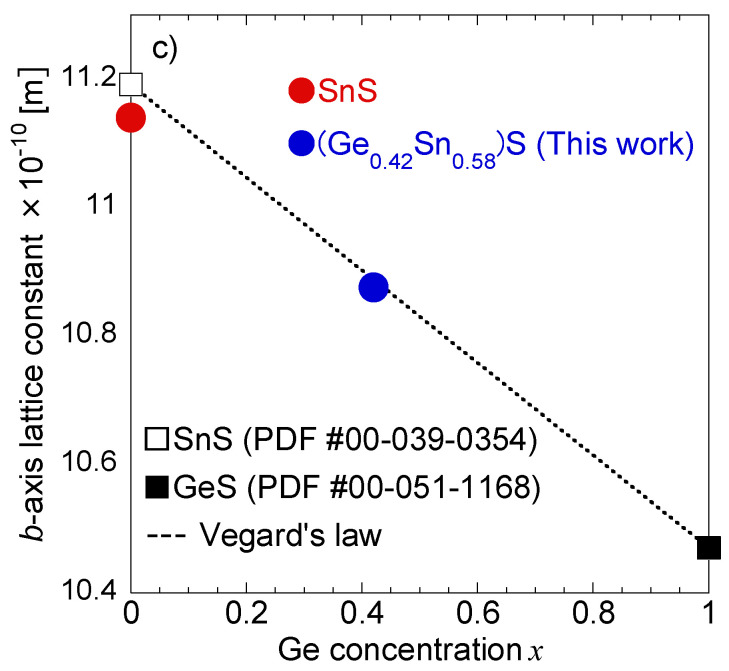
(**a**) XRD patterns of the fabricated SnS [32] and (Ge_0.42_ Sn_0.58_)S thin films. The relevant PDF cards are shown at the bottom of the figure as a reference. (**b**) Magnified views of the XRD patterns are shown in the range of 30–35° (Red line: SnS thin film, blue line: (Ge_0.42_ Sn_0.58_)S thin film). (**c**) Dependence of the *b*-axis lattice constant on Ge concentration. The lattice constant *b* is calculated from the peak assigned to the (040) plane.

Figure 2a shows the XRD patterns of the fabricated SnS and (Ge, Sn)S thin films. XRD measurements were performed using MiniFlex with a NaI scintillation counter used as a detector. This apparatus has a vertical goniometer with a radius of 150 mm and an accuracy of ±0.02°. The measurement parameters were as follows: X-ray = 30 kV/15 mA, divergence slit (DS) = variable, scattering slit (SS) = 4.2°, receiving slit (RS) = 0.3 mm, and K_β_ foil filter. The measurement range was 10–90° with a step of 0.01°. The pattern of the fabricated SnS thin film was similar to that of orthorhombic SnS, while the (Ge_0.42_ Sn_0.58_)S thin film exhibited peaks at 31.27°, 32.37°, and 32.90°, as shown in Figure 2b. These peaks could be attributed to the (101), (111), and (040) planes of a (Ge, Sn)S solid solution, or to the different phases of Sn_2_S_3_, GeS, and GeS_2_. A plot of the estimated lattice constant of the *b*-axis for the most intense peak at 32.90° is shown in Figure 2c. The estimated lattice constant b, calculated using the ICDD Powder Diffraction File for SnS and GeS (PDF SnS #00-039-0354 and GeS #00-051-1168), roughly followed the Vegard rule; thus, a (Ge, Sn)S solid solution was formed in the (Ge_0.42_ Sn_0.58_)S thin film. Moreover, as shown in Figure 2a, a peak at 12.90° (possibly due to Sn_2_S_3_) was observed in the XRD spectrum of the (Ge_0.42_ Sn_0.58_)S thin film; here, the peak appeared at a higher angle than its position in the reference (Sn_2_S_3_ #00-014-0619) spectrum, probably because a part of Sn_2_S_3_ was substituted by Ge to form (Ge, Sn)_2_S_3_.

**Figure 3:**
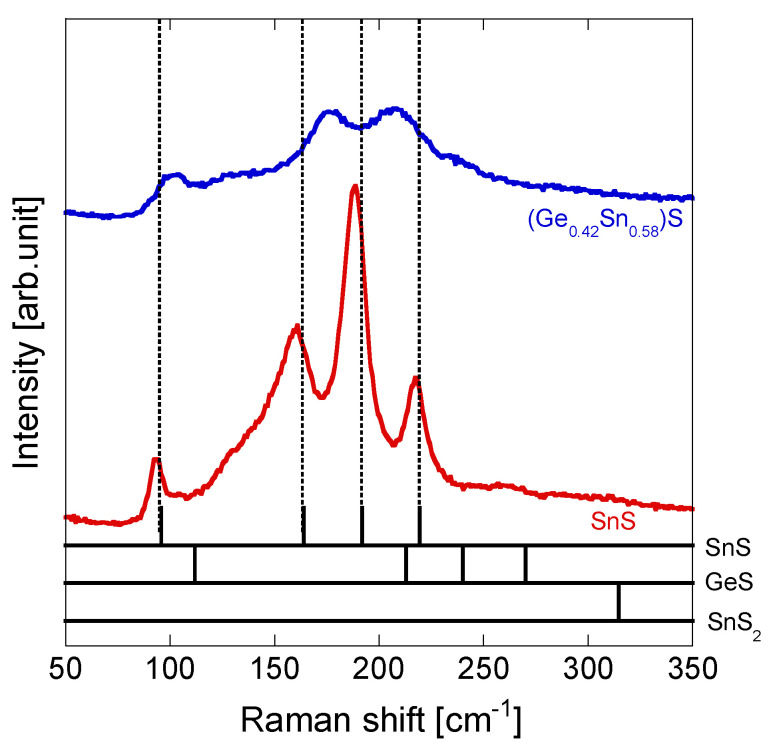
Raman spectra of the fabricated SnS [32] and (Ge_0.42_ Sn_0.58_)S (red and blue lines, respectively) thin films. The reference Raman spectra of SnS [35], GeS [36], and SnS_2_ [37] are shown by black lines at the bottom of the figure.

Figure 3 shows the Raman spectra of the SnS [32] and (Ge_0.42_ Sn_0.58_)S thin films. The spectral peaks of the SnS thin film could be attributed to orthorhombic SnS; peaks were observed at 95.9 cm^−1^ (A_g_), 164.0 cm^−1^ (B_3g_), 192.0 cm^−1^ (A_g_), and 219.5 cm^−1^ (A_g_) (the vibrational modes are indicated in brackets) [35]. No peaks attributable to GeS and SnS_2_ were observed [36,37]. A higher-wavenumber peak shift was observed in the (Ge_0.42_ Sn_0.58_)S spectrum; this could be attributed to the substitution of the Sn sites in SnS by Ge. A similar shift of Raman peaks has been reported by Araki et al. in a similar sulfide solid solution, Cu_2_(Ge*_x_* Sn_1−*x*_)S_3_, which is attributed to the substitution of Sn by Ge [38]. Therefore, a (Ge, Sn)S solid solution was formed in the Ge*_x_* Sn_1−*x*_S system in this study, even with a Ge composition corresponding to a high Ge/(Ge + Sn) ratio of 0.42. Raman spectroscopy did not indicate any of the different phases (such as GeS_2_ or Sn_2_S_3_) indicated by XRD analysis; thus, it is suggested that (Ge, Sn)S predominantly existed near the surface of the (Ge_0.42_ Sn_0.58_)S thin film.

**Figure 4:**
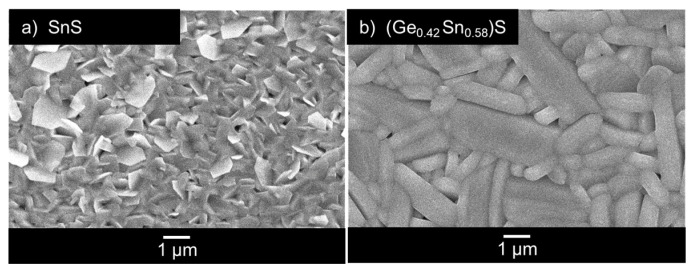
Surface morphology of (**a**) SEM images of surface morphology at different locations on the same sample as SnS in Ref. [32] and (**b**) (Ge_0.42_ Sn_0.58_)S (annealed), observed using SEM. A white scale bar is shown at the bottom of the figure.

Figure 4a shows the SEM surface morphology of SnS. Block-like and plate-like crystal grains were observed on the SnS surface; these grains are commonly observed in SnS [39]. Figure 4b shows the surface morphology of (Ge_0.42_ Sn_0.58_)S. The observed grains are elongated platelets. The observed 1–5 µm grains, which are considerably larger than those of SnS, are thought to be due to the crystal growth of microcrystal grains by annealing. Sinsermsuksakul et al. reported that as the film thickness increased, the {010} plane switched its crystallographic orientation from parallel to perpendicular to the substrate [17]. This suggests that the difference in the morphology of SnS and (Ge_0.42_ Sn_0.58_)S may be partly due to the difference in film thickness. From the aforementioned XRD results, the (Ge_0.42_ Sn_0.58_)S thin film was not preferentially oriented in one direction, but the orientation of each grain was considered to be different, as peaks that can be attributed to (111) and (040) are observed in the film. They have also pointed out that the plane perpendicular to the substrate is a desirable structure for solar cells because it significantly improves the mobility in the thickness direction compared to the plane parallel to the substrate [17]. The differences in contrast between the individual grains observed in Figure 4b may be due to differences in conductivity resulting from differences in orientation.

**Figure 5:**
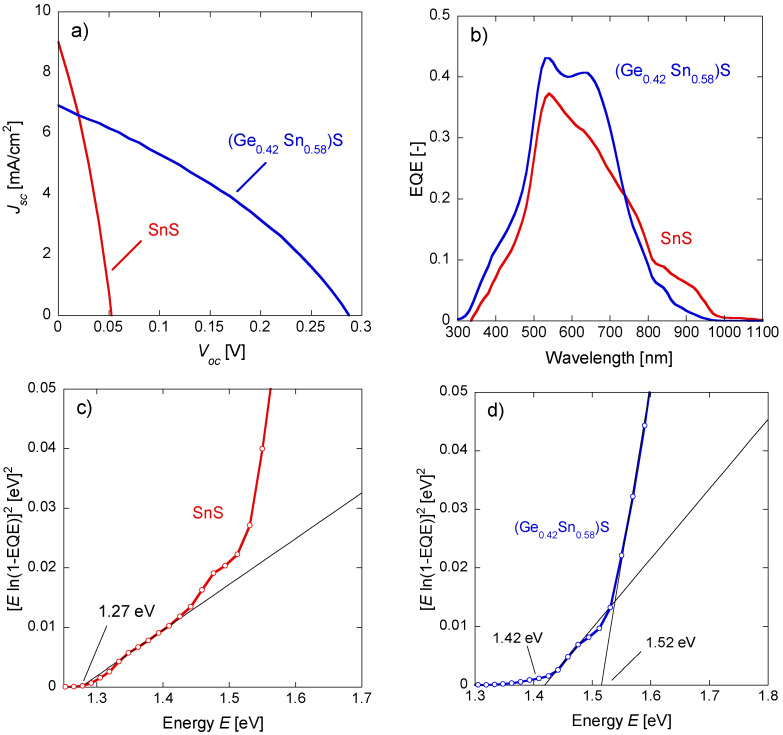
J-V curves and EQEs for the fabricated solar cells; the red and blue lines correspond to the SnS and (Ge_0.42_ Sn_0.58_)S thin-film solar cells, respectively. (**a**) J-V curves of solar cells fabricated with the SnS and (Ge_0.42_ Sn_0.58_)S thin films. (**b**) The EQEs of solar cells fabricated with the SnS and (Ge_0.42_ Sn_0.58_)S thin films. (**c**) Energy (*E*) versus [*E* ln(1-EQE)]^2^ plot and extrapolated line (black line) in the EQE plot of the SnS thin-film solar cell. (**d**) *E* versus [*E* ln(1-EQE)]^2^ plots and extrapolation lines for (Ge_0.42_ Sn_0.58_)S thin-film solar cells, similar to (**c**).

Figure 5a and Table 3 show the *J*-*V* curves and photovoltaic characteristics of SnS and (Ge_0.42_ Sn_0.58_)S thin-film solar cells. The solar cells fabricated using SnS thin films showed an open circuit voltage (*V*_oc_) of 0.053 V, short circuit current density (*J*_sc_) of 9.00 mA/cm^2^, fill factor (*FF*) of 0.32, and power conversion efficiency (*PCE*) of 0.15%. Contrarily, solar cells fabricated using the (Ge_0.42_ Sn_0.58_)S thin film showed a *V*_oc_ of 0.29 V, *J*_sc_ of 6.92 mA/cm^2^, *FF* of 0.34, and *PCE* of 0.67%. The (Ge_0.42_ Sn_0.58_)S device showed a higher *V*_oc_ than the SnS device, with similar values of *FF*. The efficient formation of a p-n junction interface between the n-type CdS and p-type (Ge, Sn)S possibly improved the *V*_oc_ of the (Ge_0.42_ Sn_0.58_)S device; the *V*_oc_ value of the device fabricated here is greater than those of previously reported solar cells based on (Ge_0.27_ Sn_0.73_)S thin films [32]. The *V*_oc_ deficit *V*_ocfef_ = *V*_oc_ − *E*_g_/*q* (V), the difference between the band gap and the open circuit voltage estimated from the absorption edge of the EQE, which will be discussed below, was *V*_ocdef_ = 1.217 V for *x* = 0 (E_g_ = 1.27 eV). For *x* = 0.42 (E_g_ = 1.42 or 1.52 eV), *V*_ocdef_ = 1.13 or 1.23 V. This large *V*_ocdef_ indicates that significant recombination occurs in both samples. As the *V*_ocdef_ is almost the same magnitude for both samples, the change in *V*_oc_ can be attributed to the increase in band gap due to the introduction of Ge. This could be due to an increase in the band gap of the optical absorption layer due to an increase in the Ge concentration and the formation of large crystal grains due to the annealing process. Furthermore, the *J*_sc_ of the solar cell based on (Ge_0.42_ Sn_0.58_)S thin films was slightly lower than that based on SnS thin films, possibly due to the presence of different phases, such as GeS_2_ and Sn_2_S_3_, which promoted recombination. Figure 5b shows the EQE of the fabricated SnS and (Ge_0.42_ Sn_0.58_)S thin-film solar cells. The highest EQE was recorded at about 530 nm for both devices, possibly because only the carriers near the light absorption layer (SnS or (Ge_0.42_ Sn_0.58_)S)/CdS interface were extracted; carriers that were excited deep inside the light absorption layer (near the Mo back electrode) underwent recombination due to the short diffusion length of the carriers, and could not be extracted. The energy E versus [E ln(1-EQE)]^2^ plots for the SnS and (Ge_0.42_ Sn_0.58_)S thin-film solar cells are shown in Figure 5c and d, respectively. The band gap of SnS was estimated to be 1.28 eV by extrapolation, similar to the previously reported value [8,9,15,21,22,23]. The (Ge_0.42_ Sn_0.58_)S spectrum shows a tail at the absorption edge, and its band gap was estimated to be in the range of 1.42–1.52 eV, indicating a non-uniform Ge concentration in (Ge, Sn)S, or absorption due to subgap defect levels.

## 4. Conclusions

This study reports the synthesis of (Ge_0.42_ Sn_0.58_)S thin films using co-evaporation. XRD and Raman spectroscopy confirmed that a (Ge, Sn)S solid solution was formed in the (Ge_0.42_ Sn_0.58_)S thin film. The open circuit voltage (*V*_oc_), short circuit current density (*J*_sc_), *FF*, and *PCE* of solar cells fabricated using the (Ge_0.42_ Sn_0.58_)S thin film were 0.29 V, 6.92 mA/cm^2^, 0.34, and 0.67%, respectively. Moreover, the bandgap of the (Ge_0.42_ Sn_0.58_)S thin-film solar cell was estimated to be 1.42–1.52 eV. In summary, in this study, we report the synthesis of a (Ge_0.42_ Sn_0.58_)S thin film with a Ge content of 0.42—confirmed to be a (Ge, Sn)S solid solution—and its application in solar cells. Among the (Ge, Sn)S solar cells reported thus far, it was clarified that solar cells can be realized even in a composition region with a relatively high Ge concentration. However, the photoelectric conversion characteristics are still inadequate, and improvements such as the suppression of thin-film crystal defects and the formation of appropriate heterojunction interfaces are required to improve the efficiency further. In addition, more detailed research on (Ge, Sn)S is required, such as the fabrication of (Ge, Sn)S thin films with even higher Ge concentrations and the realization of (Ge, Sn)S thin-film solar cells.

## Figures and Tables

**Table 1:** Film fabrication conditions for the co-evaporation process.

	SnS [32]	(Ge*_x_* Sn_1−*x*_)S
Deposition time [hours]	3	3
Ge cell temperature [°C]	-	1200
Sn cell temperature [°C]	1015	1010
S temperature [°C]	150	150
Substrate temperature [°C]	300	150
S-Valve opening	3	1.5

**Table 2:** Film thicknesses and constituent-element compositions estimated by XRF.

	Thickness [µm]	Ge/(Ge + Sn)	(Ge + Sn)/S
SnS [32]	0.687	0	0.98
(Ge*_x_* Sn_1−*x*_)S (As deposited)	1.087	0.42	0.95
(Ge*_x_* Sn_1−*x*_)S (Annealed)	1.033	0.42	0.98

**Table 3:** Photovoltaic characteristics of solar cells fabricated using the as-prepared SnS and (Ge_0.42_ Sn_0.58_)S thin films.

	Area [cm^2^]	*V*_oc_ [V]	*J*_sc_ [mA/cm^2^]	*FF*	*PCE* [%]
SnS [32]	0.1577	0.053	9.00	0.32	0.15
(Ge_0.42_ Sn_0.58_)S	0.1514	0.29	6.92	0.34	0.67

## Data Availability

Data are contained within the article.
